# A Facile Method for the Fabrication of Luminescent Eu^3+^-Doped SiO_2_ Nanowires

**DOI:** 10.3390/gels8050286

**Published:** 2022-05-06

**Authors:** Fei Gao, Xinyu Zhao, Jinglin Liu

**Affiliations:** College of Chemistry and Materials Science, Inner Mongolia Minzu University, Tongliao 028000, China; zhaoxydq@163.com (X.Z.); liujinglin@imun.edu.cn (J.L.)

**Keywords:** tartaric acid, europium, silica nanowires, sol–gel, photoluminescence

## Abstract

Europium trivalent ion (Eu^3+^)-doped silica nanowires were prepared, and the positioning of Eu^3+^ in the silicon dioxide nanowire matrix was researched. Scanning electron microscopy (SEM), transmission electron microscopy (TEM), X-ray diffraction analysis (XRD) and energy-dispersive X-ray spectroscope analysis (EDX) were used to characterize the product’s morphology and structure. The representation of Fourier transform infrared spectra (FT-IR) and X-ray photoelectron spectroscopy (XPS) were indicative of the presence of a covalent Eu-O-Si bond. The results suggest that Eu^3+^ was successfully doped into amorphous silica. Furthermore, a sol-gel inorganic−organic co-assembly mechanism model was proposed to illuminate the formation of the rare-earth ion-doped nanowires. In addition, photoluminescent emission of europium ions in a silica matrix was further discussed. It was demonstrated that a 10% content of Eu^3+^ resulted in a quenching effect and after annealing at 650 °C, the europium ions in the nanowires had a high luminescence intensity due to the silica network structure.

## 1. Introduction

For the past few years, nanostructured materials like nanotubes [1], nanorods [2], nanoparticles [3] and nanowires [4] have aroused unprecedented attention as a result of their excellent properties, including optical [5], electrical [6], magnetic [7] and catalytic [8] abilities. A fairly large number of nanostructures (i.e., nanowires (NWs)) have been synthesized by various techniques over the past decade, such as carbothermal reduction [9], thermal evaporation [10,11], vapor phase transport [12,13], bio-mimetic strategies [14], excimer laser ablation [15], thermal chemical vapor deposition [16] and the solution method [17,18].

Particularly, amorphous silica nanostructures have drawn significant attention as a broad, prospective optical material with well-known bulk properties [19]. Heretofore, SiO_2_ NWs have been synthesized using several approaches. For instance, SiO_2_ NWs were formed with the assistance of Mg [20] and Ni [21] as catalysts by thermal evaporation. In addition, rare-earth-doped SiO_2_ NWs possess some distinct and beneficial properties such as biocompatibility and photoelectricity [22], which made them become important for numerous modern technologies (i.e., biosensors [23], cell-imaging [24], organic light-emitting diodes (OLED) [25,26] etc.) Lin et al. [5] reported the preparation of terbium(III)-doped SiO_2_ nanowires based on the vapor−liquid−solid principle. The novel green emission from both doped Tb^3+^ ions and host SiO_2_ has been detected from these nanowires. Liu et al. [27] found that the luminescence intensity of Tb^3+^ was enhanced by six times when the ions were capped with SiO_2_ nanowires. Nevertheless, the research progress of rare-earth ion-doped SiO_2_ NWs is still limited.

Hereby, we report a simple method to prepare Eu^3+^-doped amorphous SiO_2_ nanowires using a sol-gel process. A growth model based on a co-assembly mechanism is discussed in detail. Moreover, according to the characterization results, it is shown that the trivalent europium ions are bound successfully in the host network in the shape of an Eu-O-Si bond.

## 2. Experimental Procedure

### 2.1. Materials and Preparation

#### 2.1.1. Materials 

Absolute ethyl alcohol, distilled water, D, L-dihydroxybutane dioic acid (TTA), ammonium hydroxide, aqua fortis (Sinopharm Chemical Reagent Beijing Co., Beijing, China, analytical grade), tetraethyl orthosilicate (TEOS) (Aladdin reagent (Shanghai) Co., Shanghai, China, analytical grade) and europium sesquioxide (Goring High-Tech Material Inc., Ganzhou, China, 4N) were used in all cases.

#### 2.1.2. Preparation of Eu^3+^-Doped Silica Nanowires 

In a typical procedure, 0.2 mmol TTA was dissolved in 5 mL H_2_O/EtOH. Under a static condition in a water bath kettle at 25 °C for 24 h, ready-prepared europium nitrate aqueous solution (0.1 mol/L) was dropped into the former solution. After that, TEOS (0.47 mL) was mixed with the solution system fleetly, whisking the whole time. An 80 μL NH_4_OH (28 wt% NH_3_ solution) was added after stirring for 5 min. After stirring for 30 min and standing for another 48 h, white precipitate was then obtained. The emulsion could be separated by centrifuging and non-ion washing three times, whereafter the milk white gel was found. Subsequently, the gelatum was shifted to a drying oven, staying at 55 °C for 24 h. This was followed by a thermal treatment (calcination) at 650 °C for 4 h with a heating rate of 1 °C/min. Finally, during spontaneous cooling to room temperature, a kind of dry white powder was formed.

### 2.2. Characterization

The research used scanning electron microscopy (SEM) (Hitachi, S-4800, Chiyoda City, Tokyo, Japan) as a means of analyzing the morphology and nanostructure of the obtained nanowires with Eu^3+^ as the dopant. An energy-dispersive X-ray (EDX) detector was operated using an H JEOL JXA-840 system (Akishima-shi city, Tokyo, Japan) installed on the SEM microscope. A JEM-2000EX transmission electron microscope (acceleration voltage of 200 kV, Akishima-shi city, Tokyo, Japan) provided accurate TEM images for detailed structural analysis. The X-ray diffraction (XRD) pattern was obtained on a Rigaku D/max-B II X-ray diffractometer (Akishima-shi city, Tokyo, Japan) using Cu Ka radiation, which was used to examine the phase state of the samples. Fourier transform infrared (FT-IR) analyses of the Eu^3+^-doped silica nanowires were carried out by a Perkin−Elmer 580B infrared spectrophotometer (Waltham, MA, USA) through the potassium bromide pellet technique. The chemical bonds in the obtained nanowires with Eu^3+^ as the dopant were characterized through X-ray photoelectron spectroscopy (XPS) (Thermo Fisher Scientific, ESCALAB250, Waltham, MA, USA). The photoluminescence (PL) spectra were obtained using a Jobin Yvon FluoroMax-4 luminescence spectrophotometer (Paris, France) (excitation source was a 150 W xenon lamp). All the tests were undertaken at 25 °C.

## 3. Results and Discussion

### 3.1. Characterization of Samples

**Figure 1:**
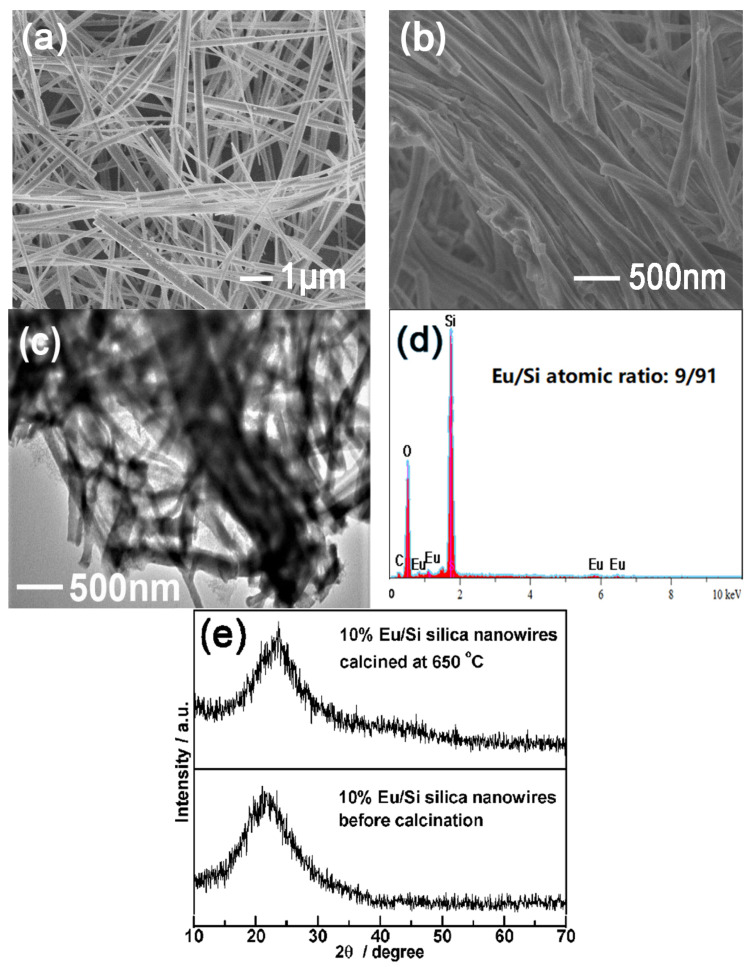
Trivalent europium ion-doped silica nanowires obtained (Eu/Si molar ratio is 10%) at 25 °C: (**a**) SEM image without calcination; (**b**) SEM image of calcinated sample; (**c**) TEM image of calcinated sample; (**d**) EDX patterns of calcinated sample; (**e**) XRD patterns.

In our previous studies, the nanofibers formed spontaneously in Eu^3+^-tartrate solution at 25 °C had good uniformity and dispersibility with several micrometers length and 150–300 nm diameter [28]. In this procedure, TEOS is added in the europium salt of the organic acid system at the initial stage of the nanostructure-forming process, which means the process of organic nanowire growth and silicon dioxide condensation takes place almost at the same time. After the hydrolyzation and polycondensation of TEOS in the presence of ammonia, silica nanowires are prepared. SEM images before (Figure 1a) and after calcination (Figure 1b) show a large mass of vast Eu^3+^-doped silica nanowires several microns long. From TEM image (Figure 1c), it can be observed that nanowires with solid construction can be maintained after calcination, and their diameters are in the range of 50~200 nm.

An EDX diagram of calcined Eu^3+^-doped silica nanowires (Figure 1d) demonstrates that the nanowires are composed of three elements: Eu, O and Si. The atomic ratio of Eu/Si is 9:91, nearing to an addition of 10% of Eu^3+^. In addition, the slight peak of carbon observed in the pattern is probably derived from a small quantity of carbon-based material (labeled in the lower left corner of Figure 1d).

Figure 1e shows an XRD spectrogram of Eu^3+^-doped silica nanowires obtained with and without thermal treatment at 650 °C. The wide peak at 2θ = 22.5° can be assigned to the characteristic diffraction of the amorphous silicon dioxide according to JCPDs, and the peak intensity of the calcined sample decays gradually and fluctuates slightly [29]. Moreover, there is no europium-related phase observed, which indicates that Eu^3+^ has entered the silica lattice.

### 3.2. Factors Influencing the Reaction

To get the optimal reaction conditions of silica nanowires with trivalent europium ions as the dopant, much more involved research has been conducted. SEM was applied to investigate the morphology of the nanowires.

**Figure 2:**
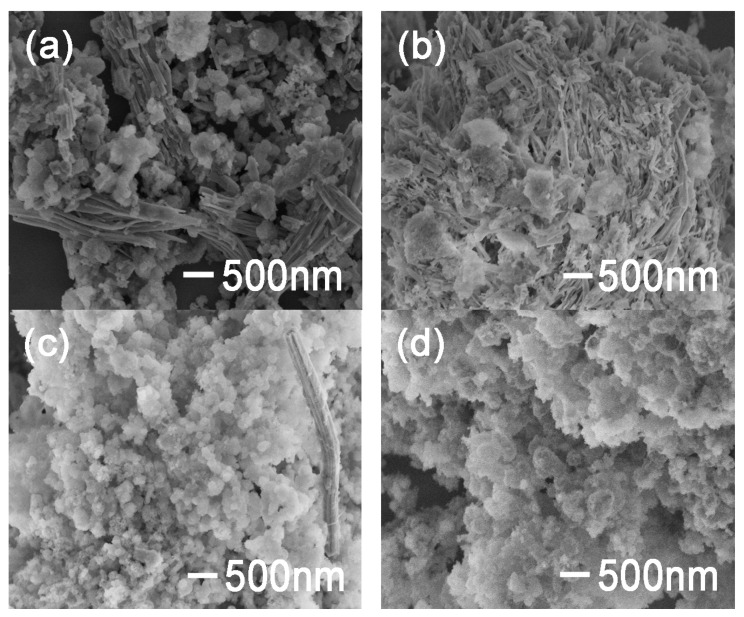
SEM images of Eu^3+^-doped silica nanowires with different dosages of H_2_O: (**a**) 2 mL; (**b**) 4 mL; (**c**) 8 mL; (**d**) 10 mL.

First, Figure 2 reveals a series of typical SEM photographs of the sample under different amounts of solvent, confirming that the product obtained using 5 mL deionized water (Figure 1b) has a more uniform morphology in length (several microns) and diameter (50~200 nm) than the others. More aggregations emerged with fewer amounts of water (Figure 2a,b), probably because the relatively high concentration of ammonia impels TEOS to hydrolyze rapidly. TTA molecules disperse unevenly or have insufficient time to self-assemble. On the contrary, excess solvent (Figure 2c,d) makes the interaction between the trivalent europium ion and the TTA too weak to form nanowires.

**Figure 3:**
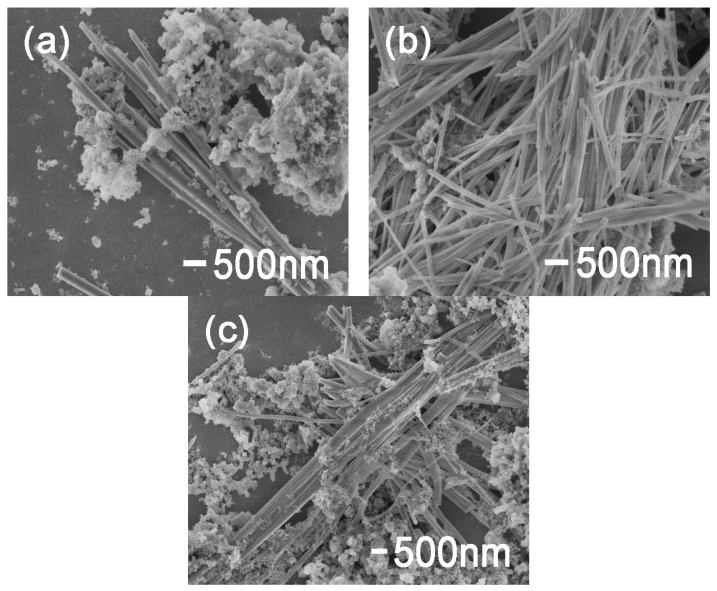
SEM images of Eu^3+^-doped silica samples obtained at different standing times of the solution: (**a**) 10 h; (**b**) 24 h; (**c**) 48 h.

Secondly, the sol process before TEOS addition was investigated to confirm the optimal standing time (Figure 3b). It is noted that a standing time above or below 24 h is not beneficial for the formation of nanowires, which is mainly reflected in the morphology. As shown in the corresponding SEM image (Figure 3a), the system did not have enough time to complete co-assembly after only standing for 10 h. In addition, Eu^3+^-TTA formed spontaneously by self-assembly when the standing time increased to 48h. That means silica oligomers may not be a part of the nanowire structure (Figure 3c).

### 3.3. Formation Mechanism

**Figure 4:**
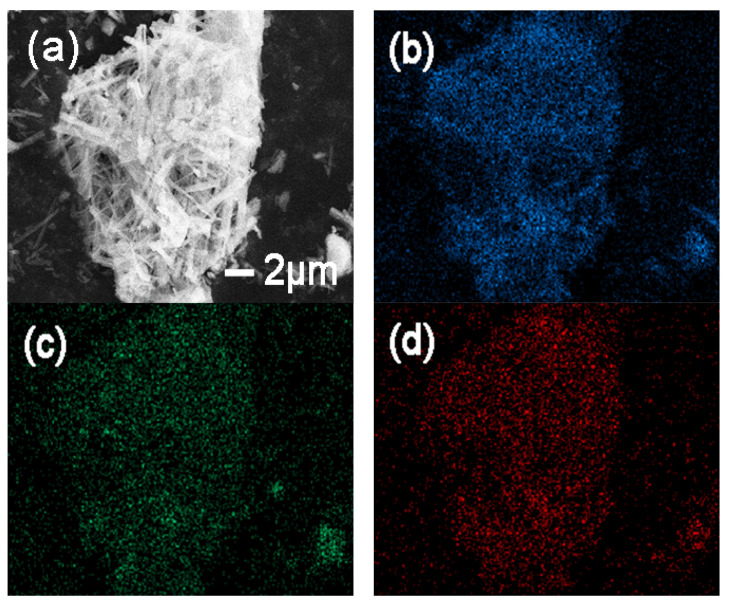
SEM-EDX elemental distribution maps. (**a**) SEM image of Eu^3+^-doped nanowires; (**b**) distribution map of O; (**c**) distribution map of Si; (**d**) distribution map of Eu.

At first, EDX elemental mapping of Eu^3+^-doped silica nanowires was used to identify the distribution of O, Si and Eu (Figure 4a–d) after the calcination treatment. As seen in Figure 4b,c, Si and O were distributed equally in accordance with the nanowire morphology. Figure 4d revealed that europium was distributed in the structure of silica nanowires uniformly. In summary, it is preliminarily indicated that Eu^3+^ embedded in the silica network.

**Figure 5:**
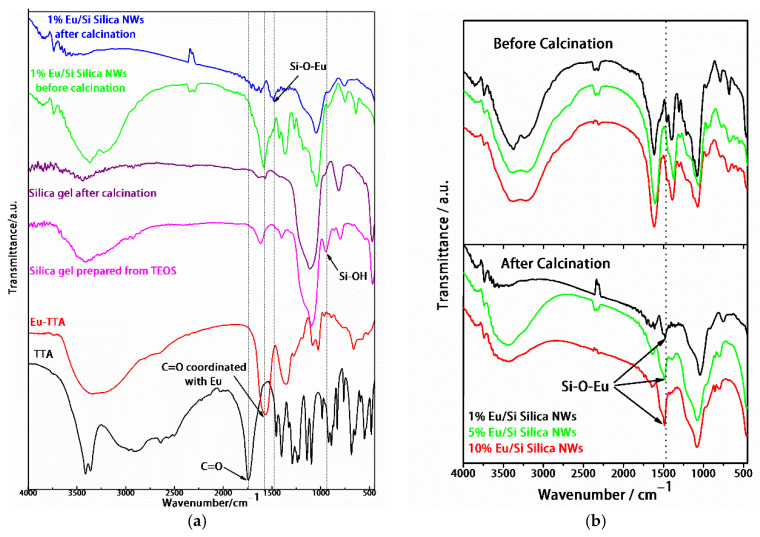
FT-IR spectra of TTA, Eu^3+^-tartrate, silica gel (before and after calcination), 1% Eu/Si SiO_2_ NWs (before and after calcination) (**a**) and Eu^3+^-doped SiO_2_ NWs prepared in different doping content at 25 °C before and after calcination (**b**).

Secondly, an infrared spectrogram was recorded to provide evidence of the formation of Eu^3+^-doped silica networks after calcination. The symmetrical stretching and bending vibrational modes of the Si-O bond can be identified at 805 and 467 cm^−1^. The peak located at 945 cm^−1^ due to Si–OH vibration was clear in silica gel but showed some intensity in silica nanowires before calcinations, and almost disappeared after thermal treatment in both (Figure 5a). This may mean that Si–OCH_2_CH_3_ in TEOS doesn’t hydrolyze to Si–OH when switching to Si–O–Si networks completely, but likely combines with the hydroxy from TTA in the silica NW reaction system. Likewise, it was observed that the peak at 1100 cm^−1^ on account of the asymmetric Si-O-Si vibrational stretching mode is broader and stronger in calcinated samples. The broad peak (3415 cm^−1^) is mainly attributed to the hydroxyls in the free water molecules in the sample without calcination, except for the terminal hydroxyl groups, which turn weak after calcination [30,31].

Additionally, the band at 1743 cm^−1^ corresponds to the stretching vibrations of the C=O bonds of carboxylate ions in pure TTA. The red shift of this band to 1616 cm^−1^ in the Eu^3+^-tartrate complex’s spectrum is ascribed to the coordination between Eu^3+^ and C=O bonds in TTA [32,33], which also appear in the Eu^3+^-doped silica nanowires before calcination. It is indicated that Eu ions get into the NW’s reaction system before calcination in the same way as the Eu^3+^-tartrate complex. Interestingly, the carboxylate bands disappear after thermal treatment, and a new spiculate and prominent band appears at 1486 cm^−1^ in the spectrum of Eu^3+^-doped silica nanowires after calcination, which is found nowhere else in Figure 5a and is assigned to the Eu-O-Si bond [34,35]. With the rise of rare-earth trivalent ion-doping content, the significant increase in the characteristic band near 1486 cm^−1^ (Figure 5b) is indicative of the fact that high-temperature calcination induces the formation of Si–O–Eu, which shows that europium ions are doped in the silica matrix.

**Figure 6:**
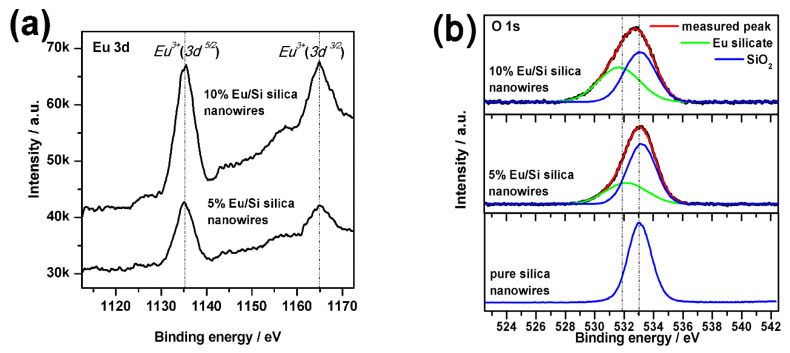
XPS spectra of (**a**) Eu 3d and (**b**) O 1s for Eu^3+^-doped SiO_2_ nanowires in different doping content after calcination.

Thirdly, XPS was carried out to characterize the doped silica nanowires. The spectra of europium 3d and oxygen 1s levels of nanowires are shown in Figure 6. In the europium 3d (5/2) spectrum (Figure 6a), the main peak at 1135.2 eV (binding energy) matches Eu^3+^, while the peak located at about 1164.8 eV originates from europium 3d (3/2) of trivalent europium ions as well.

The oxygen 1s spectra, with their appropriate peak curve-fitting lines for the pure and doped silica nanowires after calcinations, are shown in Figure 6b. Each fitting peak followed the general shape of the Gaussian function. The low energy state at 531.9 eV is attributed to the O atoms in Si–O–Eu. The high energy state at 533.1 eV is assigned to the O in SiO_2_ [36]. Furthermore, the intensity of the O 1s peak in accordance with silicate gradually enhances with increasing Eu^3+^, while the O 1s peak intensity weakens slightly in accordance with SiO_2_. Compared to the pure amorphous silica nanowire’s reference position, the O 1s peak of 10% Eu/Si silica nanowires is shifted to a lower binding energy by about 0.3 eV, indicating more Eu–O–Si structures are formed in the higher Eu concentration samples [37]. This result further indicates that trivalent rare-earth ions penetrate the amorphous silicon dioxide.

**Scheme 1:**
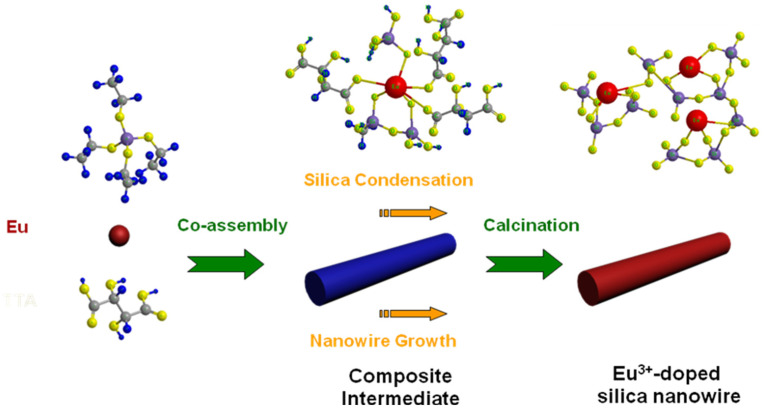
Possible mechanism of composite intermediates and Eu^3+^-doped SiO_2_ nanowire formation.

According to the previous characterization results, a probable co-assembly mechanism model could be proposed for the synthesis of Eu^3+^-doped SiO_2_ nanowires (as shown in Scheme 1), which contains three steps: (1) Eu ions coordinate with the TTA carboxyl by self-assembly in an aqueous solution to form nanofibers. The morphology, bound together by hydrogen bonding and metal coordination, should be stably maintained during the sol-gel reaction [38]; (2) Meanwhile, TEOS subsequently hydrolyzed into anionic oligomeric silica species. These oligomeric groups are easily attracted to relatively acidic HC-OH groups in TTA due to electron withdrawing of the two carboxylates. These groups can act as “acids” to exchange an ethoxy group in TEOS, with the formation of triethoxysilyl groups on the Eu-TTA complex chain. These triethoxysilyl groups may be condensed upon increasing the pH, with NH_4_OH adding and making a three-dimensional silica nanowire network with homogeneous Eu distribution. The growth of nanowires and the process of silica polycondensation proceed simultaneously. Hence, europium ions, D, L-TTA, and anionic oligomeric silica species co-assemble into inorganic−organic hybrid nanowires [39]; (3) Finally, the products are obtained after the organic template is removed by washing and calcination. When Eu^3+^-tartrate nanofibers are destroyed, europium ions are successfully doped in the amorphous silica in the shape of Eu-O-Si.

### 3.4. Photoluminescence Properties

**Figure 7:**
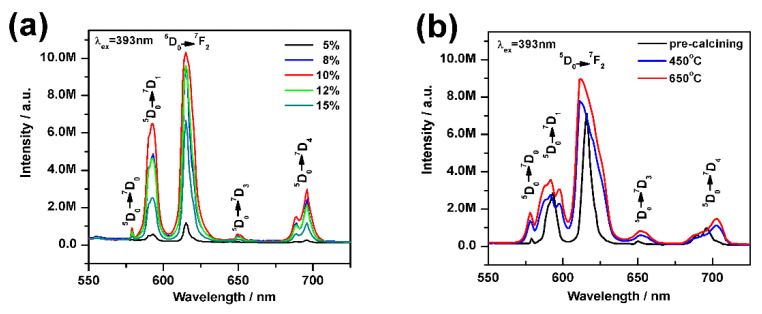
PL emission spectra of Eu^3+^-doped SiO_2_ samples (excitation wavelength is 393 nm) prepared at 25 °C with (**a**) different doping contents of Eu^3+^ and (**b**) different calcination temperatures.

The photoluminescence emission spectra of Eu^3+^-doped SiO_2_ nanowires in different dosage concentrations (molar ratio Eu^3+^: SiO_2_) by ultraviolet light excitation (393 nm) are shown in Figure 7a. All phanic peaks match with the transitions from the metastable orbital singlet state ^5^D_0_ to the spin–orbital states of ^7^F_J_ (J = 0, 1, 2, 4) of the europium trivalent ion [40], which suggests the characteristic transitions from ^5^D_0_ to ^7^F_0_ (578 nm), ^7^F_1_ (587 nm, 592 nm and 597 nm), ^7^F_2_ (613 nm), ^7^F_3_ (650 nm) and ^7^F_4_ (700 nm). The intensity of the emission peak at 613 nm enhances gradually with increasing Eu^3+^ concentration. The peak reaches its strongest point at 10% doping content of Eu^3+^ and gradually reduces when the dopant is added sequentially. This could probably be attributed to the concentration-quenching effect of Eu^3+^.

The Eu^3+^ emission spectra recorded for the synthesized 10% Eu^3+^-doped SiO_2_ nanowires obtained by calcination at 450 °C and 650 °C are displayed in Figure 7b. The annealed spectral shapes were obviously different from the Eu^3+^ in the synthesized nanowires, indicating the relaxation process from dissimilar electron levels of the Eu^3+^ because of the matrix. The site environment of Eu^3+^ results in the significant luminescence enhancement of the Eu^3+ 5^D_0_→^7^F_2_ [41]. The presence of -OH groups before calcination could reduce the luminous efficiency of Eu^3+^ due to a non-radiative phonon quenching [42]. Therefore, the luminescence intensity of Eu^3+^-doped SiO_2_ nanowires annealed at 650 °C, with the luminescence intensity of the entire SiO_2_ framework being higher than the synthesized samples after annealing at 450 °C.

## 4. Conclusions

Europium trivalent ion-doped SiO_2_ nanowires were synthesized, and a sol-gel inorganic−organic co-assembly mechanism was proposed. During the formation process, Eu^3+^-tartrate is likely to cooperate with silicate groups and enters the silica nanowires. The situation of the rare-earth ions in the silica network structures was investigated. With the doping, significant changes in the characteristic FTIR peaks of Eu-O-Si and XPS spectra were observed. In addition, photoluminescent emission of Eu^3+^ in a silica matrix was researched by adjusting the doping contents and calcination temperatures. It was found that the quenching concentration of Eu^3+^ is at 10% and Eu^3+^-doped SiO_2_ nanowire annealed at 650 °C, with the entire SiO_2_ framework having a high luminescence intensity on account of the site environment of Eu^3+^.

## Data Availability

Not applicable.
